# Puerperal Fever

**Published:** 1844-12

**Authors:** Robert Storrs

**Affiliations:** Doncaster


### PRACTICAL MEDICINE, &c.

*Puerperal Fever.*—By Robert Storrs, Esq., Doncaster.

[We publish the substance of Mr. Storr's paper, not so much to prove the contagiousness of certain forms of puerperal fever, respecting which opinion there can be very little doubt, but to show the mode by which the practitioner may carry the contagion from one patient to another: and we would particularly call the attention of every obstetric practitioner to this interesting subject. Mr. Storrs thinks that medical men do not go far enough in considering this disease to be propagated by medical men and nurses from one puerperal patient to another. He thinks that it is quite as frequently carried by the medical attendant to each fresh labor-case from some original infectious case, whether of gangrenous erysipelas, or typhus fever, or of whatever animal poison besides may hereafter be found to produce it. In some cases which occurred in his own practice, he has no doubt that he took it to each patient from a case of gangrenous erysipelas with subsequent abscess, which he was attending at the time of these unfortunate occurrences. And such may be the case of other practitioners. They think that they convey the contagion from one puerperal patient to another, instead of from one common source. They probably lose a puerperal case and immediately take every precaution to prevent a similar occurrence, by careful ablution, and a complete change of dress. Nevertheless the next case of labor is attended with the same fatal result; simply because the practitioner is still in attendance on the case which originally gave rise to the mischief. Mr. Storrs was not only himself convinced of this fact, but wished to ascertain the experience of other practitioners, and for this purpose he wrote to several of those who had met with the disease in their practice. Among the rest to Mr. Reedall, of Sheffield, from whom he received the following remarks.]

At the time of my attendance on those females who were subsequently attacked, I had under my care a young man laboring under sloughing bubo, combined with erysipelatous inflammation of the scrotum and nates, of a malignant character, which required dressing daily, and which ultimately proved fatal. It may be somewhat corroborative of the supposed dependence of this form of puerperal fever on an animal poison generated by this sore, and propagated by contact, that the sister of the young man, who waited upon him, was seized with erysipelas of the head and face, of a very low, typhoid nature, which terminated fatally in a few days.

It would be unnecessary in me to repeat my implicit belief in the contagiousness of this disease, and its connection with this case of erysipelas; but if further confirmation were needed, I might adduce the circumstance, that immediately antecedent to my taking the charge of the above case of erysipelas, I had met with no case of puerperal fever, and that upon discontinuing my attendance upon the young man (which I immediately did upon the belief that I was the medium of conveying infection from him to the puerperal cases), I had no recurrence of the puerperal fever.

I may give it as my opinion, that in all my cases the disease had one common origin, viz., the bubo, and was not communicated from case to case. I should also wish to state that at the same time those cases occurred to me, Mr. Parker, then my pupil, but now resident in Sheffield, attended many midwifery cases, and all recovered well. He never visited the erysipelatous patient. The above cases were not confined to one locality, but were living in different parts of the town, showing that the disease did not arise from any local cause. As I have previously stated, I had very strong suspicion, after the first case or two, that I was conveying the infection, but could not discover how until the sister was seized. This was the most malignant case of erysipelas I ever witnessed. I then began to think whether I was not conveying the poison from this source.

[In these as well as in all other cases reported, treatment proved unavailing. Mr. Storrs proceeds to say—]

Three surgeons, residing in the same town, attended the post-mortem examination of a patient who had died from gangrene after an operation for strangulated hernia, and were all of them employed in handling the diseased parts. One of them was called from the inspection to a case of labor, which terminated in fatal puerperal fever; he had others in rapid succession. The other two surgeons had also fatal cases of puerperal fever within a day or two after the same inspection. On casually meeting, they mentioned their misfortunes to each other, and were thus convinced of the origin of the disease. They all abandoned practice for a short period, and had no more of it. I would here briefly draw the attention of the reader to the remarkable and striking fact of Mr. Reedal having five fatal cases of this horrid malady in labors which he attended so immediately after dressing a case of malignant erysipelatous disease, and on his leaving off attendance on this case having no more of it, and that neither his pupil even, nor any other medical gentleman in Sheffield, had any instances of it among their labor cases; also that Mr. Sleight had two cases whilst in attendance on a case of erysipelas; that Mr. Hardey's cases also arose while he was in attendance on a case of sloughing abscess and of erysipelas. And again, that three surgeons were simultaneously the means of spreading puerperal fever from one post-mortem examination of a case of gangrenous erysipelas—a combination of evidence I think sufficient to convince the most sceptical that this disease produces a subtle animal poison, which is instrumental in propagating, when puerperal women are subjected to its influence whose predisposition favors it, a disease in about thirty-six or forty-eight hours afterwards of the most inflammatory, prostrating, and violent character—a disease which stamps death on the features and in the symptoms immediately on its occurrence.

[Mr. Storrs next enumerates a great many cases recorded by different practitioners, all of which prove that each disease had a common origin from some case of erysipelas or sloughing ulcer.

He concludes his very interesting paper with the following advice:]

As it is well to be always guarded against such a misfortune, I think it desirable for midwifery practitioners to avoid attending labors in the same dress in which they attend their ordinary patients, especially the coat, as this garment must be the one most likely to be the means of conveying fomites; and at any suspicious period, when typhus or erysipelas are prevailing, to carry out the same carefulness even in the after-attendance on labor cases. I should also, after a post-mortem of any kind, or after any operation upon any case of erysipelas, or of typhus, recommend the most careful ablutions of the hands, and for the surgeon to avoid attending on a labor in any part of the dress in which such operations have been performed, not forgetting the gloves, as the hand and arm are the chief instruments of contact. Where, however, the disease has been unfortunately once set up in a practice, an absence from home for a fortnight or three weeks, a total change of raiment, the most careful ablutions, and a perfect avoidance of every case likely to have been the source of animal poison, should alike be adopted by the practitioner.—*Prov. Med. Journal.*

[In the Provincial Journal for January 13, 1844, p. 287, Mr. Elkington, of Birmingham, relates several cases which in a remarkable degree confirm the previous facts and opinions.]—*Braithwaite's Retrospect.*

